# Screening of Neutralizing Antibodies Targeting Gc Protein of RVFV

**DOI:** 10.3390/v17040559

**Published:** 2025-04-12

**Authors:** Chuanyi Zhao, Meng Hao, Ting Bian, Xiaofan Zhao, Xiangyang Chi, Zhengshan Chen, Guangcheng Fu, Zheng Zhu, Ting Fang, Changming Yu, Jianmin Li, Wei Chen

**Affiliations:** 1 School of Medicine, Zhejiang University, Hangzhou 310058, China; chuanyizhao@126.com; 2 Laboratory of Advanced Biotechnology, Beijing Institute of Biotechnology, Beijing 100071, China; haorm_23@126.com (M.H.); bianting@126.com (T.B.); xiaofan_zhao@foxmail.com (X.Z.); xiangyangchi@163.com (X.C.); czs0076@163.com (Z.C.); fgc8s9s@126.com (G.F.); zhuzheng0627@163.com (Z.Z.); 18612006386@163.com (T.F.); yuchangming@126.com (C.Y.)

**Keywords:** Rift Valley fever virus (RVFV), neutralizing antibody (NAb), Gc protein, VHH, bispecific antibody (bsAb), nanobody (Nb)

## Abstract

Rift Valley fever virus (RVFV) is a mosquito-transmitted bunyavirus that can cause substantial morbidity and mortality in livestock and humans, for which there are no currently available licensed human therapeutics or vaccines. Therefore, the development of safe and effective antivirals is both necessary and urgent. The Gc protein is the primary target of the neutralizing antibody response related to Rift Valley fever virus. Here, we report one Gc-specific neutralizing antibody (NA137) isolated from an alpaca and one bispecific antibody (E2-NA137), the protective efficacies of which we evaluated in A129 mice. In this prophylactic study, the survival rates of the NA137 and E2-NA137 groups were both 80%, and in the treatment study, the survival rates were 20% and 60%, respectively. Altogether, our results emphasize that NA137 and E2-NA137 provide a potential approach for treating RVFV either prophylactically or therapeutically.

## 1. Introduction

Rift Valley fever (RVF) is a mosquito-borne viral zoonosis caused by Rift Valley fever virus (RVFV), an important pathogen that was first isolated in the Great Rift Valley in Kenya in 1930 [1]. Pregnant ruminants such as cattle, camel, and especially sheep are highly susceptible to RVFV infection and are typically subject to high rates of febrile illness, fetal malformation, and abortions, and newborn lambs usually show nearly 100% mortality [2,3]. The clinical symptoms caused by RVFV infection can differ according to the severity of the infection and include fever, headache, orbital pain, muscle pain, severe encephalitis, visual disturbance, jaundice, hepatitis, bleeding, and potentially death [4]. From 1950 to 1998, epidemics causing thousands of deaths were reported on the African continent, such as in South Africa, Kenya, Egypt, and some countries in East Africa [5]. The first reported occurrence of the spread of Rift Valley fever (RVF) outside of the African continent was in Saudi Arabia and Yemen in September 2000 via the trading of infected livestock, resulting in the deaths of hundreds of people [6,7]. In 2016, China reported its first imported case, a patient returning from Angola, a non-epidemic country [8]. These cases demonstrate the potential for the cross-border transmission of RVFV. At present, no licensed vaccines or antiviral drugs have been approved for use in humans [9]; thus, the development of preventive vaccines and specific therapeutic drugs is crucial for the prevention and control of this disease.

The taxonomy of bunyaviruses has evolved drastically since 2016, with the promotion of the Bunyaviridae family to the Bunyavirales order [10,11]. Bunyaviruses possess a common genetic organization, consisting of a segmented negative or ambisense RNA genome composed of small (S), medium (M), and large (L) genome segments, with the M segment encoding the virion glycoproteins Gn and Gc. The bunyavirus glycoproteins Gn and Gc form spikes on the lipid bilayer envelope of the virion and facilitate the viral invasion of a host cell. Indeed, numerous studies have demonstrated that bunyaviruses are transported into the low-pH endosomal lumen via different endocytosis pathways. The fusion of the virion and the endosome, which is triggered following the exposure of the virion to a low-pH environment, has been shown to trigger conformational changes in the glycoproteins of many bunyaviruses. The structure of the phenuivirus Gc ectodomain has been determined for RVFV, SFTSV, and Heartland virus (HRTV) [12,13,14,15]. In contrast to the phlebovirus Gn, the Gc protein is relatively conserved. Gc consists of three domains (designated as I, II, and III), with the fusion loop located in domain II, which is critical for insertion into the target membrane of the host cell. The fusion loop-directed monoclonal antibody induced via natural CCHFV infection possesses both neutralizing activity and a certain degree of neutralizing breadth [16]. Daniel Wright et al. reported that the Gn N-terminal region occupies the most membrane-distal region of the virus, effectively shielding the cognate Gc [17].

RVFV is a negative single-stranded RNA virus belonging to the genus Phlebovirus under the family Phenuiviridae in the order Bunyavirales [1]. RVFV contains a tripartite RNA genome consisting of large (L), medium (M), and small (S) segments [18]. The L segment encodes viral RNA-dependent RNA polymerase (RdRp), which synthesizes both viral mRNA and genomic RNA [19], the S segment encodes a nonstructural protein (NS protein) and nucleoprotein (N protein), and the M segment encodes the viral glycoproteins Gn and Gc [18]. The Gn and Gc proteins exist as heterodimers on the surfaces of virus particles and are important components for viral attachment and membrane fusion, respectively [12,14,20]. The Gc protein exhibits the same topology as that of its counterpart, which is similar to that of another Phlebovirus member: severe fever with thrombocytopenia syndrome virus (SFTSV) [21,22,23]. Gn and Gc proteins are the primary targets for the development of neutralizing antibodies and vaccines related to Rift Valley fever virus [24,25,26], and several studies have shown that the generation of neutralizing antibodies against Gn and Gc provides a good correlate of protection in a variety of animals, such as mice, rhesus macaques, and sheep [27]. Because the Gc protein is shielded by the Gn protein before the virus binds to host cells, as previously reported [17,23], it is more conserved and easier to screen for broad-spectrum neutralizing antibodies against RVFV compared with the Gn protein. In 2019, Qihui Wang et al. isolated one Gc-specific monoclonal antibody (R5) from a convalescent RVF patient [28]. Subsequently, a related study in 2021 showed that five human neutralizing monoclonal antibodies targeting the Gc protein were selected [29]. However, no licensed therapeutic antibodies targeting Gc antigens have been approved for use in humans. Therefore, effective antivirals are urgently needed.

Interestingly, in camelids and nurse sharks, heavy-chain-only antibodies (HCAbs) are found, of which the variable domain of the heavy chain (VHH) can be expressed as a single-domain antibody (sdAb) or nanobody (Nb) [30]. The VHHs derived from camelid heavy-chain antibodies are 10-fold smaller than those of conventional antibodies, with a molecular weight of about 15 kDa, and exhibit several advantageous features [31,32]. VHHs are intrinsically highly soluble molecules, and due to their distinctive structure with an extended antigen-binding CDR3 region and overall small size, some can bind epitopes that are inaccessible to conventional antibodies (e.g., cryptic functional epitopes, such as the virus-binding site of a cell surface receptor) [33]. Moreover, VHHs with nanomolar or even picomolar affinities target a broad spectrum of antigens, as previously described [4,34]. Their single-domain nature additionally allows for easy genetic manipulation, which can endow VHHs with additional effector functions, e.g., extended in vivo half-lives, cytotoxicity, or translocation through the blood–brain barrier [35,36,37]. VHHs have broad application prospects in the biomedical, diagnostic, and therapeutic fields, and there have been numerous studies on the antiviral activities of nanobodies against various viruses, including human immunodeficiency virus type 1 (HIV-1) [38], Chikungunya virus (CHIKV) [39], MERS CoV [40], and SARS-CoV-2 [41]. The FDA approved the first bivalent nanoantibody, caplacizumab, for the treatment of acquired thrombotic thrombocytopenic purpura in 2018 [42]. In September 2022, ozoralizumab, the first nanobody compound, was approved in Japan for the treatment of rheumatoid arthritis (RA) [43].

Bispecific antibodies (bsAbs) is the term used to describe a large family of molecules designed to recognize two different epitopes or antigens [44]. BsAbs, also known as second-generation antibodies, have stronger specificity and targeting, fewer adverse reactions, and the ability to overcome the escape mechanism in treatment compared with monoclonal antibodies. Therefore, they are widely used and have broad application potential in the treatment of various diseases. Bispecific antibodies that engage two distinct viral epitopes have the potential to provide enhanced potency and breadth [45]. For instance, some bispecific antibodies against viral infections have shown neutralizing activities against a variety of viruses, including HIV [46], influenza viruses [47], Ebola viruses [47], dengue virus [48], and Crimean–Congo hemorrhagic fever [16]. BsAbs come in many formats, ranging from relatively small proteins to large conventional antibodies [44]. As previously reported [49], bsAbs have been developed for SARS-CoV-2, such as the IgG -VHH format of SYZJ001 [50], the DVD-Ig format of CV1206_521_GS [51], and IgG-like FD01 [52].

In this study, we describe a chimeric VHH antibody targeting the Gc protein, NA137, which was isolated from an alpaca immune library using phage display and fused with human Fc. The neutralizing activity of NA137 against RVFV was assessed, with an IC_50_ value of 2.701 μg/mL. To enhance the potential neutralizing activities of antibodies, we engineered bispecific antibodies in the IgG-VHH format, named E2-NA137, E2-NA19, and E2-NA220. Subsequently, we evaluated the protective efficacies of NA137 and E2-NA137 against the RVFV challenge in an interferon-α/β receptor-deficient A129 mice model. In summary, NA137 and E2-NA137 are candidate therapeutic agents for Rift Valley fever.

## 2. Materials and Methods

### 2.1. Ethics Statement and Biosafety Information

All animal experiments were approved by the Animal Welfare and Ethics Committee of the Academy of Military Medical Sciences (permit number: IACUC-SWGCYJS-2021-006; date of approval: 11 March 2021). The mouse RVFV challenge experiments were conducted in animal biosafety level 2 (ABSL2) facilities at the Academy of Military Medical Sciences, Beijing, China.

### 2.2. Cells, Viruses, and Animals

Vero E6 cells were cultured in Dulbecco’s modified Eagle’s medium (DMEM) (Gibco, Grand Island, NY, USA) supplemented with 10% fetal bovine serum (FBS) (Gibco, Grand Island, NY, USA), 100 U/mL penicillin, and 100 mg/mL streptomycin at 37 °C with 5% CO_2_. The rescued viruses rMP-12 and rMP-12-eGFP were prepared as previously described [53], propagated in Vero E6 cells, and stored at −80 °C. Interferon-α/β receptor-deficient (IFNAR(-/-)) A129 mice were housed and bred in the animal facility of the Animal Center, Beijing Institute of Biotechnology (Beijing, China). One adult alpaca (#459) was purchased and housed at Abiocenter Biotechnology Co., Ltd. (Wuxi, China).

### 2.3. Gc Protein Expression and Purification

The RVFV Gc ectodomain (residues 691–1119) was subcloned into a pCAGGs vector. The C-terminal of the plasmid was labeled with strep-tag II. Subsequently, the plasmid was transfected into Expi293F cells, and then the recombinant Gc protein was purified using a StrepTrapTM HP column (Cytiva, Uppsala, Sweden) in an AKTA Pure purification system (GE Healthcare, Piscataway, NJ, USA).

### 2.4. Immunization of Alpaca

The mRNA-Gc_691–1119_ was prepared as previously described [54] and diluted with PBS. The adult alpaca (#459) was intramuscularly (hind leg) immunized with 2 mL of mRNA-Gc_691–1119_ (500 μg) four times, with an interval of 14 days. Additionally, alpaca serum was collected after the fourth immunization. An enzyme-linked immunosorbent assay (ELISA) was used to detect the specific antibody titer against Gc in the serum, with pre-immunization serum used as a negative control. After determining that a high antibody titer had been obtained, the blood sample was taken for library preparation.

### 2.5. Construction of Single-Domain Antibody Library

A week post the fourth vaccination, a blood sample was collected for the library preparation, and peripheral blood mononuclear cells were harvested for total RNA isolation and cDNA synthesis. After the amplification of the nanobody-coding gene by two rounds of PCR, the PCR products were purified, digested with the SfiI restriction enzyme, and cloned into the appropriately cut phagemid vector pComb3X. The recombinant phagemid was introduced into competent XL1-Blue cells via electroporation, and phages displaying the VHH library were produced and used immediately in phage display panning.

### 2.6. Panning of the Gc-Specific Phage Display Nanobody Library

Next, 96-well plates (9018, Costar, Washington, DC, USA) were coated overnight with 5 μg/mL RVFV Gc protein in carbonate–bicarbonate buffer (pH 9.6) at 4 °C, washed with PBST (0.1% Tween 20 in PBS), blocked with 3% skimmed milk powder, and incubated at room temperature for 1 h. Subsequently, the plates were washed with PBST, and the nanobody library was added to the blocked 96-well plates and incubated at 37 °C for 2 h. After washing with PBST, the bound phage was eluted with glycine–HCl and then neutralized with Tris–HCl to a pH of 7.4. Next, the competent XL1-Blue cells were infected with the eluted phage and incubated at 37 °C for 30 min, followed by centrifugation. The precipitate was suspended in a fresh medium and cultured on plates. After the fourth or fifth round of panning, individual clones were picked and used for further identification.

### 2.7. Screening of Gc-Specific Positive Clones

The 0.5 μg/mL Gc protein was coated onto 96-well ELISA microtiter plates overnight, and 3% skimmed milk powder was coated as the negative control. After blocking with 3% skimmed milk powder, the supernatant expressed by the phages was added and incubated at room temperature for 1 h. The 96-well plates were washed with PBST, and then HRP-labeled M13 monoclonal antibodies (100 µL per well) were added at 0.2 µg/mL and incubated at room temperature for 1 h. Then, the plates were washed with PBST, and TMB was added (200 µL per well). The reactions were stopped by a stop solution (50 µL per well), and the absorbance was measured at 450 nm and 630 nm using a microplate reader. An OD value greater than 0.5 and an OD value of the Gc antigen group greater than three times the OD value of the negative control group were regarded as positive clones.

### 2.8. Cloning, Expression, and Purification of VHH-Fc

After the fourth or fifth round of panning and transduction to XL1-Blue cells, individual colonies were picked. The CDR3 sequences of VHH were compared, and unique sequences were screened with Genous software. The positive clones were used as templates to amplify the gene fragments of the VHH fragments, and the amplified gene was cloned into the pCDNA3.4 expression vector via EcoRI and EcoRV restriction enzyme sites. Subsequently, the constructed plasmids that encoded the VHH–hFc fusion protein were expressed using Expi293F cells and were then purified using a HiTrap rProteinA purification column (Cytiva, Uppsala, Sweden) in an AKTA Pure purification system (GE Healthcare, Piscataway, NJ, USA). The molecular weights and purities of the antibodies were confirmed through sodium dodecyl sulfate–polyacrylamide gel electrophoresis (SDS-PAGE).

### 2.9. Enzyme-Linked Immunosorbent Assays (ELISAs)

The 96-well plates (9018, Costar, Washington, DC, USA) were coated overnight with 2 μg/mL of the RVFV Gc protein in carbonate–bicarbonate buffer (pH 9.6) at 4 °C and were then washed with PBST (0.2% Tween 20 in PBS) and blocked with 2% BSA in PBS at 37 °C for 1 h. The plates were washed three times with PBST, and serum samples from the immunized alpaca or antibodies were serially diluted and added to the blocked plates. After incubation at 37 °C for 1 h, the plates were washed with PBST and incubated with Goat Anti-Alpaca IgG H&L (HRP) (Catalog No. S001H, AlpVHHs, Chengdu, China) and HRP-labeled Goat Anti-Human IgG Fc (Catalog No. ab97225, Abcam, Cambridge, UK) at 37 °C for 1 h. Then, the plates were washed with PBST, TMB substrate solution (Catalog No. PR1200, Solarbio, Beijing, China) was added to the microplates for 6 min at room temperature, and the reactions were terminated by a stop solution (Catalog No. C1058, Solarbio, Beijing, China). The absorbance at 450 nm and 630 nm was read using a microplate reader (SPECTRA, Molecular Device, San Jose, CA, USA). We defined the endpoint titers as the reciprocal of the highest serum dilution, the OD value of which was 2.1-fold higher than that of the negative control.

### 2.10. Virus Neutralization Tests

The neutralizing activity of the alpaca serum was measured as previously described [55,56]. Serum samples were heated at 56 °C for 30 min before use. The 3-fold serial dilutions of the serum or antibodies were mixed and incubated with the same volume of rMP-12-eGFP (100 TCID_50_/well) in 96-well plates at 37 °C for 1 h. A total of 20,000 Vero E6 cells in 100 μL of DMEM supplemented with 5% FBS were subsequently added to each well and cultured at 37 °C with 5% CO_2_ for 48 h. Cell controls (without virus and antibodies) and virus controls (with virus only) were set up. After 48 h, the supernatant was discarded, and the cells were fixed with 4% paraformaldehyde overnight. Following this, the cells were washed with PBS three times and immune-stained with a DAPI counterstain (Thermo Scientific, Waltham, MA, USA). The infected cell count (eGFP) and total cell count (DAPI) were detected using the Celigo (Nexcelom, Boston, MA, USA) imaging cytometer. We defined the focus of the reduction neutralization test (FRNT_50_) as the reciprocal of the serum dilution that inhibits 50% of the viral infection compared with virus-only wells. The infection value was selected, and the percent neutralization of the antibodies was calculated as 100% − (sample values − cell control values)/(virus control values − cell control values) × 100%. A three-parameter nonlinear regression analysis was performed, and the IC_50_ values of the antibodies were calculated using GraphPad Prism 9 (GraphPad Software, Inc., San Diego, CA, USA).

### 2.11. KD Analysis via the Biacore System

The Biacore™ T200 System (Cytiva, Shanghai, China) was used to determine the dissociation constant (KD) of VHH-hFc or IgG-VHH, based on surface plasmon resonance (SPR). The RVFV Gc protein was diluted to 0.5 μg/mL using Hepes-Buffered Saline (HBS)-EP+ buffer (Catalog No. BR100669, Cytiva) and fixed to the protein A sensor (Catalog No. 29127555, Cytiva). After that, VHH-hFc or IgG-VHH were diluted in 200 nM, 100 nM, 50 nM, 25 nM, 12.5 nM, 6.25 nM, and 3.125 nM through Hepes-Buffered Saline (HBS)-EP+ buffer and were associated with the sensors. Subsequently, dissociation steps and regenerations were performed. Finally, the data were analyzed using Biacore™ T200 Evaluation Software 3.2 to calculate the KD value.

### 2.12. Reduced SDS-PAGE

The sizes and purities of the antibodies were analyzed via SDS-PAGE and Coomassie staining. The 6 × protein loading buffer K489 (TransGen, Beijing, China) was mixed with 5 μg of each antibody for the reduced SDS-PAGE. Samples were heated for 5 min at 100 °C and loaded on 4–12% SDS gradient gel (GenScript, Nanjing, China), which was run at 180 V for about 1 h, and Coomassie staining was performed.

### 2.13. Engineering and Production of Bispecific Antibodies in IgG-VHH Format

For engineering the IgG-VHH, the VHH was fused to the C-terminus of the E2 heavy chain with the GGGGSGGGGSGGGGS linker to generate a modified IgG-VHH heavy-chain plasmid. Then, we co-transfected the IgG-VHH heavy-chain plasmid and E2 light-chain plasmid at a 1:1 ratio into Expi293F cells to generate the IgG-VHH. After 5 days of culturing, culture supernatants containing antibodies were harvested and purified using a HiTrap rProteinA purification column (Cytiva, Uppsala, Sweden) in an AKTA Pure purification system (GE Healthcare, Piscataway, NJ, USA).

### 2.14. RVFV Challenge Experiments

For the RVFV challenge experiments, the A129 mouse was selected as the animal model, and the experiments were conducted in animal biosafety level 2 (ABSL2) facilities at the Academy of Military Medical Sciences, Beijing, China. Female or male A129 mice aged 5–7 weeks were randomly divided into E2, NA137, E2-NA137, and PBS groups (*n* = 5). For all studies, mice were intraperitoneally administered with 200 μg of antibodies [29,57]. In the pre-exposure setting, the A129 mice were injected with either E2, NA137, or E2-NA137 antibodies, followed by the challenge with 1 × 10^4^ TCID_50_ of RVFV (the rMP-12 strain). In the post-exposure setting, a single dose of antibodies was intraperitoneally administered on the first day post-infection. Mice were monitored daily for their survival and body weights for 14 days post-infection, and their hearts, livers, spleens, lungs, kidneys, and brains were collected at necropsy for the histopathology assay, during which the brains, livers, and spleens were also used for immunohistochemistry staining and viral load detection. Mice exhibiting weight losses of over 20% of their initial weights on day 0 were euthanized.

### 2.15. Histopathology and Immunohistochemistry

The tissues of the A129 mice were collected and fixed in a 4% paraformaldehyde solution at RT for 24 h, embedded in paraffin, and sectioned. Hematoxylin and eosin (H&E) staining was used to identify microscopic lesions. The RVFV distribution was detected using an RVFV Gn protein-specific monoclonal antibody (E2) via immunohistochemistry.

### 2.16. Quantitative RT-PCR

The viral loads in the brains, spleens, and livers of the challenged mice were determined via qPCR. An amount of 30 mg of the samples was collected and homogenized, and the viral RNA was extracted with an RNeasy Mini Kit (Qiagen, Dusseldorf, North Rhine-Westphalia, Germany) following the manufacturer’s instructions. The total RNA amount was measured using a spectrophotometer, and all the RNA samples were uniformly diluted to 400 ng/μL prior to the reverse transcription, which was conducted using PrimeScript™ RT Master Mix (Takara, Dalian, Liaoning, China). The qPCR was performed using TaqMan™ Universal Master Mix II (Thermo Scientific, Waltham, MA, USA) with the following primers and probe, as described previously: forward primer: 5′-GAAAATTCCTGAAACACATGG-3′; reverse primer: 5′-ACTTCCTTGCATCATCTGATG-3′; probe: FAM-CAATGTAAGGGGCCTGTGTGGACTTGTG-BHQ1-3′ [58]. Reactions were run on a QuantStudio 3 instrument (Applied Biosystems, Thermo Scientific, Waltham, MA, USA). The plasmid, including a partial sequence of the RVFV L gene, was serially diluted and used to generate the standard curve. We set the threshold of the CT value to 35. If the obtained value was lower than the threshold, it was judged as positive; if it was higher than the threshold, it was judged as negative.

### 2.17. Analysis of Binding Antibody Sequences

Abalign software(Version 1.2.9) was used to analyze the sequences of antibodies with binding activities, including the CDR3 sequence length and the Seqlogo plot by entropy.

### 2.18. Simulation of Structure of Antigen–Antibody Complexes Through Alphafold3

The M gene sequence of the MP-12 strain of Rift Valley fever virus (GenBank: DQ380208.1) was obtained from the GENBANK gene sequence database (https://www.ncbi.nlm.nih.gov/genbank/ accessed on 1 February 2025), and in this study, the head region (154–469 aa) and Gc protein (691–1119 aa) of the Gn protein were extracted for analysis. The amino acid sequences of the monoclonal antibody E2 heavy chain and light chain, as well as the amino acid sequence of the nanobody NA137 heavy chain, were acquired simultaneously. The Gn antigen protein sequence, the heavy- and light-chain sequences of the monoclonal antibody E2, the Gc antigen protein sequence, and the heavy-chain sequence of the nanobody NA137 were input into the locally deployed AlphaFold 3.0.0 software for the subsequent prediction of the structure of the antigen–antibody complexes. Default parameters were used in the structural prediction process, and various confidence indicators provided by AlphaFold 3.0.0 were used to evaluate the prediction model. The local confidence score (pLDDT), prediction alignment error (PAE), prediction template modeling score (pTM), and interface prediction template modeling score (iPTM) were comprehensively evaluated. The model with the highest-ranking score was selected as the final structure, and ChimeraX 1.8 software was used for the visualization analysis to display the interaction interface and spatial conformation between antigens and antibodies.

### 2.19. Statistical Analyses

All statistical analyses in this study were performed using GraphPad Prism 9.0 software (version 9.0, GraphPad Inc., San Diego, CA, USA). Data on binding ability (ELISA) and neutralization are presented as the mean ± standard deviation (SD). One-way ANOVA with Dunnett’s multiple comparisons test was carried out for the measurement of viral loads. * *p* < 0.1, ** *p* < 0.01, *** *p* < 0.001, and **** *p* < 0.0001.

## 3. Results

### 3.1. Construction and Panning of Gc-Specific Phage Display Nanobody Library

**Figure 1:**
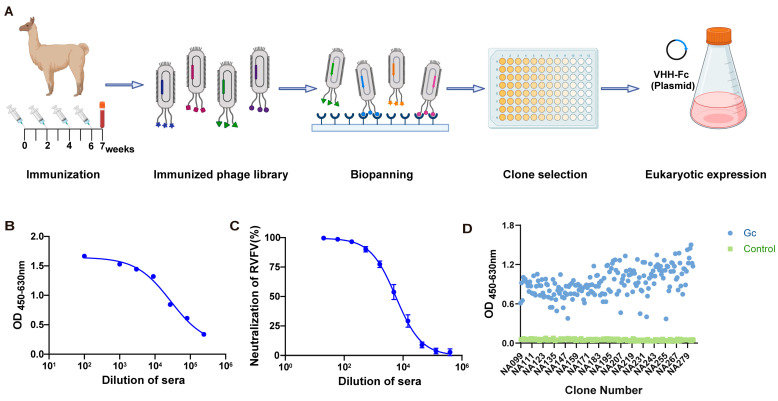
Construction and panning of the Gc-specific Phage Display Nanobody Library. (**A**) Schematic of phage-displayed VHH (variable domain of the heavy chain of heavy-chain antibodies) antibody screening and expression. (**B**) Detection of Gc-specific immunoglobulin G (IgG) titer in alpaca serum using an enzyme-linked immunosorbent assay (ELISA). (**C**) Detection of serum-neutralizing activity in immunized alpaca. (**D**) Monoclonal antibody identification from the fourth- and fifth-round screenings.

The alpaca was immunized with Gc-mRNA-LNP, and the immunization flow chart is shown in Figure 1A. Serum was collected after four rounds of immunizations, and antibody titers in the alpaca serum were detected via ELISA. The ELISA results showed that the antibody titers increased after immunization and reached 354,460 after four immunization rounds (Figure 1B). A neutralization experiment was further conducted to evaluate the neutralization activity in the immunized alpaca serum. The immunized alpaca serum showed obvious neutralizing activity against RVFV-SeGFP-infected Vero E6 cells, indicating that Gc-mRNA-LNP could stimulate high antibody levels in alpaca, providing data support for the subsequent screening of neutralizing antibodies targeting the RVFV Gc protein (Figure 1C). After four rounds of immunization, alpaca peripheral blood mononuclear cells (PBMCs) were isolated to extract the RNA fragments encoding the VHH. The amplified VHH-encoding fragments were then cloned into the pComb3X vector and electrotransformed to construct the VHH library. To obtain specific antibodies against the RVFV Gc protein, the library was screened with the Gc antigen coated with a high-affinity enzyme plate. After five rounds of panning, the clones that were specifically bound to the Gc protein were effectively enriched. We obtained 92 and 94 positive clones in the fourth and fifth rounds of screening, respectively (Figure 1D). Finally, the CDR3 sequences of the VHH were compared using Geneious software (Version 4.8.3), and 107 unique sequences were obtained. These VHH genes were further cloned into pCDNA3.4.

### 3.2. Generation and Screening for Gc-Specific Neutralizing Antibodies

**Figure 2:**
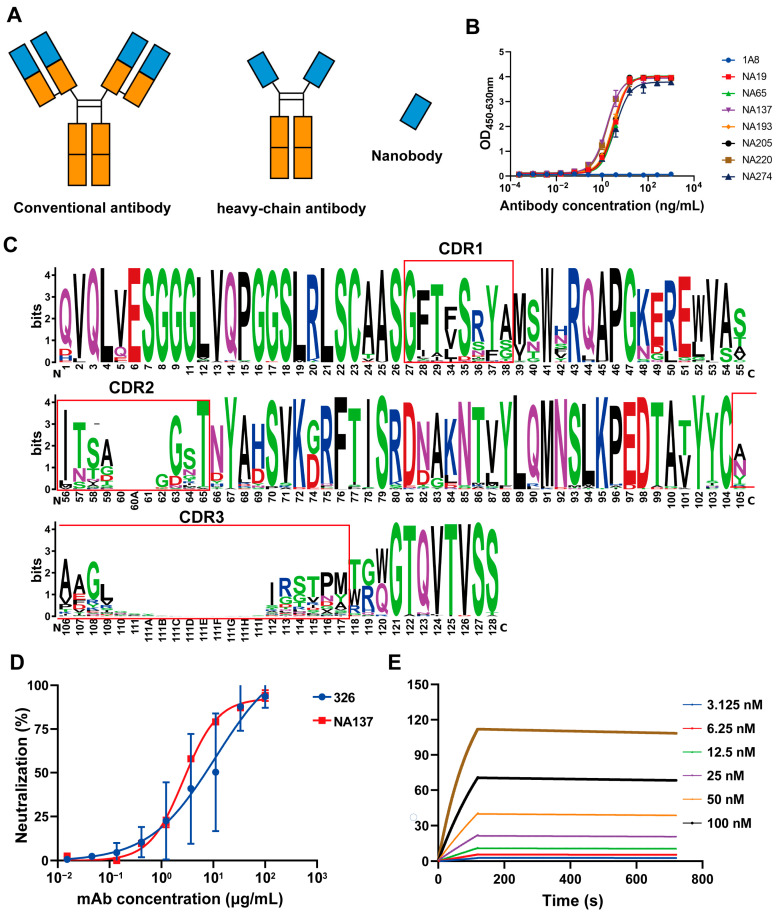
Screening for Gc-specific neutralizing antibodies and antibody sequence analysis targeting Gc protein. (**A**) Schematic comparison of nanobodies (Nbs) from heavy-chain antibodies and single-chain variable fragments (scFv) from conventional antibodies. (**B**) Binding activity of antibodies to Gc protein analyzed via ELISA. (**C**) seqLogo plot by entropy. The y-axis represents the entropy of amino acids, and the x-axis represents the position in the variable domain. The larger the letter of the amino acid, the greater the entropy value. (**D**) Protection efficacy of monoclonal antibodies (NA137) against RVFV infection in Vero E6 cells. (**E**) Determination of affinity between NA137 and Gc protein using surface plasmon resonance (SPR).

The VHH gene fragment was cloned into the pCDNA3.4 eukaryotic expression vector containing the constant-region gene of the human Fc fragment, and the VHH-hFc fusion protein expression plasmid was constructed (Figure 2A). These chimeric heavy-chain antibodies were purified using the AKTA purification system, and their molecular weights were determined using SDS-PAGE at approximately 40 kDa under reducing conditions, which were as expected. To further determine the antibody binding activity, the 96-well plates were coated with the Gc protein, and the binding capacities of the antibodies were detected through ELISA. As shown in Figure 2B, the candidates exhibited strong binding with Gc antigens. ELISA results showed that 91 antibodies had good binding activity to the Gc protein of Rift Valley fever virus, and the EC_50_ values of 65 antibodies among these antibodies were less than 10 ng/mL (Figures S1 and S2). The amino acid sequences of the antibodies were further analyzed, and most of the CDR3 region lengths were between 10 and 12 amino acids (Figure S3). In the seqlogo plot by entropy, it can be seen that the FR1, FR2, FR3, and FR4 sequences are relatively consistent, while the differences in the antibody sequences are mainly shown in the CDR1, CDR2, and CDR3 regions, among which the differences in the CDR3 region are greater (Figure 2C).

We tested the neutralizing activity of all binding antibodies and selected one nanobody (NA137) with high neutralizing activity, with an IC_50_ value of 2.701 μg/mL, as well as two nanobodies (NA19 and NA220) with weak neutralizing activity, both with IC_50_ values greater than 10 μg/mL (Figure S4). Notably, 326 was a neutralizing antibody targeting the Gc protein, as previously reported [29], and its IC_50_ value was 11.11 μg/mL (Figure 2D), indicating that the obtained NA137 antibody has neutralizing activities. Then, SPR was employed to further verify the affinity of the NA137 antibody, revealing strong binding with the Gc protein of RVFV. NA137 exhibited a high affinity at the nanomolar level, and its KD value was 1.21 nM, showing a very high affinity (Figure 2E). Overall, the NA137 antibody exhibited good neutralizing activity and high affinity, indicating its suitability for continued pharmacological development.

### 3.3. Design and Enhanced Neutralizing Potency for IgG-VHH

**Figure 3:**
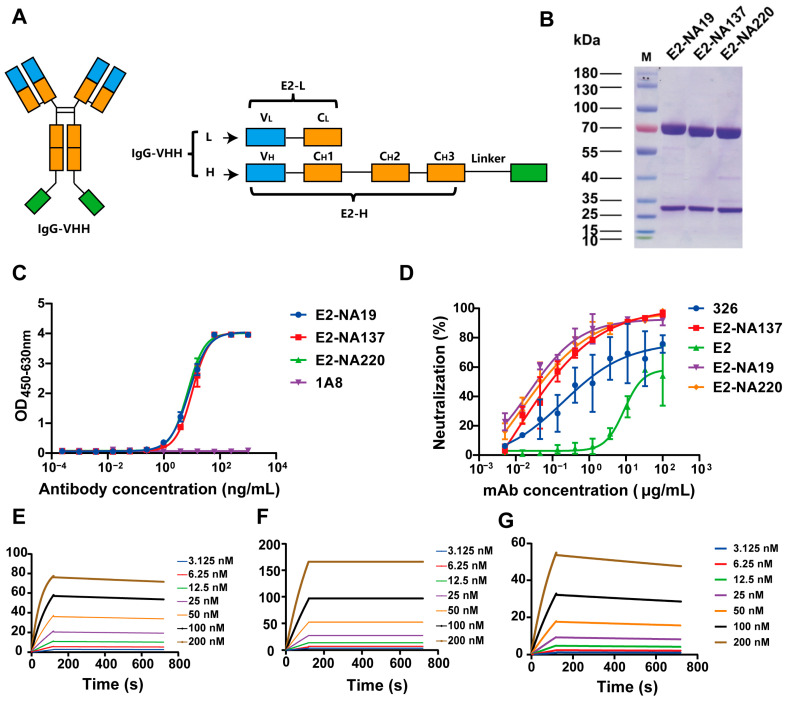
Design and enhanced neutralizing potency for IgG-VHH. (**A**) Schematic diagram of bispecific antibody. (**B**) Reduced SDS-PAGE analysis of bispecific antibody. (**C**) Binding of E2-NA19, E2-NA137, and E2-NA220 to Gc protein analyzed via ELISA. 1A8, a monoclonal antibody to the Marburg virus GP protein, was used as a negative control. (**D**) Protection efficacies of bispecific antibodies against RVFV infection in Vero E6 cells. (**E**) Determination of affinity between E2-NA19 and Gc protein using surface plasmon resonance (SPR). (**F**) Determination of affinity between E2-NA137 and Gc protein using surface plasmon resonance (SPR). (**G**) Determination of affinity between E2-NA220 and Gc protein using surface plasmon resonance (SPR).

To improve the potency of the RVFV VHHs, we engineered three IgG-VHHs. The binding antibody E2 targeting the Gn protein was selected as the parental antibody, and the VHH gene fragments of the neutralizing antibody NA137 and the neutralizing antibodies NA19 and NA220 with certain neutralizing activity were fused with a linker and named E2-NA137, E2-NA19, and E2-NA220, respectively (Figure 3A). These three bispecific antibodies were assembled at the expected molecular weights and showed good purities (Figure 3B).

The binding activities of the three bispecific antibodies were determined via ELISA. Among them, the E2-NA19, E2-NA137, and E2-NA220 EC_50_ values were 7.929 ng/mL, 10.170 ng/mL, and 7.004 ng/mL, respectively (Figure 3C). We performed neutralization assays with rMP-12-eGFP, and E2-NA19, E2-NA137, and E2-NA220 could effectively neutralize RVFV, with IC_50_ values between 0.010 and 0.023 μg/mL. Remarkably, the neutralizing activity of E2-NA137 was the best (IC_50_ = 0.010 μg/mL), and compared with NA137 (IC_50_ = 2.701 μg/mL), the neutralizing ability was significantly improved (Figure 3D).

The E2-NA19, E2-NA137, and E2-NA220 affinities to the RVFV Gc protein were determined based on the Biacore instrument (Figure 3E–G). These three bispecific antibodies exhibited strong binding with the Gc protein and were not easily dissociated. The E2-NA19 and E2-NA220 affinities were high at 1.15 nM and 5.84 nM, respectively. Notably, E2-NA137 exhibited the highest affinity at 0.00489 nM.

### 3.4. NA137 and E2-NA137 Prevented Infection When Administered Prior to Challenge

The A129 mouse is a common animal model for studying virology, immunology, and pathology, and for screening antiviral medicines and vaccines for many viruses, such as Zika virus and RVFV [53]. Therefore, the A129 mouse was selected to explore the in vivo protection efficacy of antibodies against the RVFV challenge. Based on the results of the neutralization experiment (IC_50_ = 0.010 μg/mL) and affinity (KD = 0.00489 nM), we chose E2-NA137 for the subsequent animal experiments.

**Figure 4:**
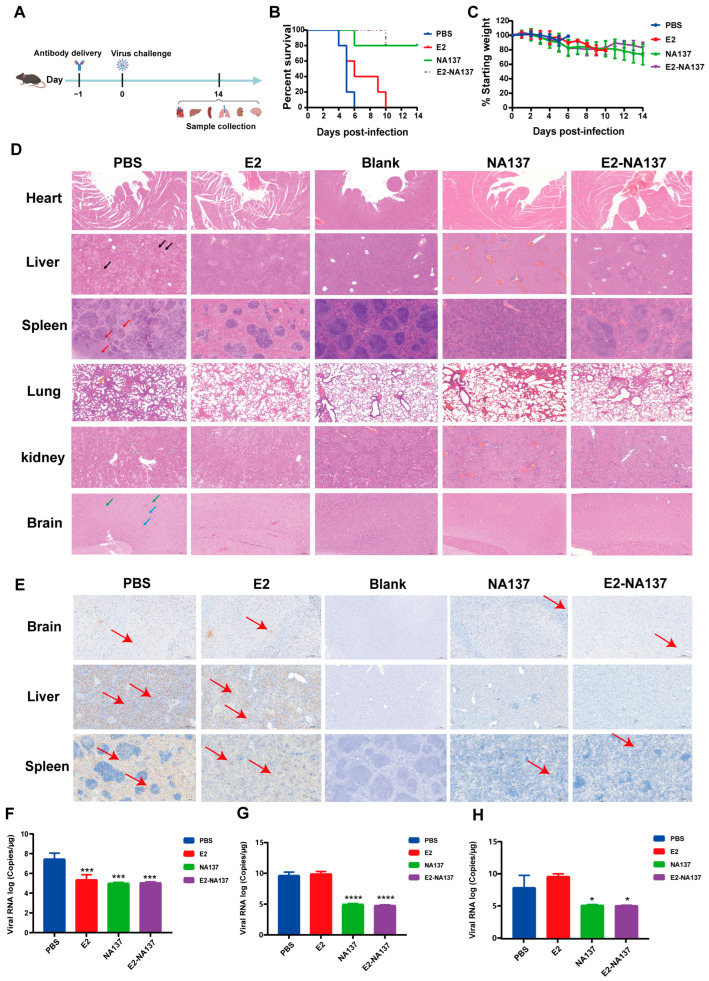
NA137 and E2-NA137 prevented infection when administered prior to the challenge. (**A**) Schematic diagram of antibody delivery, virus challenge, sample collection, and immunological assays. (**B**) Mouse mortality and survival curves. (**C**) Body weight changes in mice after RVFV challenge. The tissues from the PBS and E2 groups (at the time of death) and antibody-administrated groups (14 days after challenge) were collected and analyzed with histopathological and immunohistochemical assays. The Blank group consisted of female A129 mice that had not been challenged with RVFV and that were injected with an equal volume of PBS. (**D**) Hepatocyte necrosis is indicated by black arrows, excessive cell necrosis in the red pulp is indicated by red arrows, neuronal necrosis is indicated by green arrows, gliocyte proliferation is indicated by blue arrows, inflammatory cell infiltration is indicated by orange arrows, and necrosis of renal tubular epithelial cells is indicated by gray arrows. Representative histopathology (H&E) of hearts, livers, spleens, lungs, kidneys, and brains in RVFV-infected mice. Scale bar, 200 μm. (**E**) Representative immunohistochemistry (IHC) of brains, livers, and spleens with RVFV Gc-specific monoclonal antibodies. Red arrows indicate RVFV-positive signals. Scale bar, 200 μm. Viral loads in brains (**F**), livers (**G**), and spleens (**H**) of challenged mice were determined via qRT-PCR at the time of death (PBS and E2 groups) or 14 days after the challenge (Blank, NA137, and E2-NA137 groups). Data are displayed as means ± SD. Comparisons based on one-way ANOVA with Dunnett’s multiple comparisons test with * *p* < 0.1, *** *p* < 0.001, and **** *p* < 0.0001.

To further explore the in vivo protection efficacies of NA137 and E2-NA137 against the RVFV challenge, groups of A129 mice (*n* = 5) intraperitoneally administered 10 mg/kg of E2, NA137, and E2-NA137 at a 1-day interval were challenged with 1 × 10^4^ TCID_50_ of RVFV (the rMP-12 strain). The body weight and survival condition of each mouse were recorded daily for 14 days after the RVFV challenge (Figure 4A). At the end of the 14-day observation period, both the NA137 (200 μg) and E2-NA137 (200 μg) groups had an 80% survival rate and little change in their body weights (Figure 4B,C).

Compared with the PBS group, NA137 and E2-NA137 had relatively good preventive and protective activities in the A129 mice after the challenge. The histopathological examination indicated severe microscopic liver lesions in the mice of the PBS group at the time of death, characterized by scattered necrotic foci, inflammatory cell infiltration, and hepatocyte steatosis. Scattered splenic nodule atrophy and lymphocyte necrosis were observed in splenic tissues. Diffuse granulocyte infiltration and necrotic cellular debris were noted in the lungs, whereas primarily neuronal contraction and degeneration were noted in the brains. A large number of tubular epithelial cells in the renal cortex and medullary tissue were necrotic and exhaled, nucleopytosis was deeply stained, and a small number of eosinophilic substances were found in the renal tubule and cystic lumina. No abnormalities were discovered in the cardiac tissue, myocardial fibers, or cardiomyocytes. Moreover, there were no obvious pathological changes observed in the NA137 or E2-NA137 groups at the end of the experiment (Figure 4D). Immunohistochemical assays detected many RVFV-positive signals in the PBS group, whereas no RVFV was detected in the livers, spleens, or brains of the NA137- or E2-NA137-administered mice (Figure 4E).

At 14 days after the challenge, the surviving mice in the NA137 and E2-NA137 groups were euthanized, and the hearts, livers, spleens, lungs, kidneys, and brains were collected for histopathology assays, during which the brains, livers, and spleens were also used for immunohistochemical assays and viral RNA load analysis. The main target tissues were the brain, with an average value of 4.69 × 10^7^ copies/μg RNA, liver (7.93 × 10^9^ copies/μg RNA), and spleen (5.86 × 10^8^ copies/μg RNA) at the time of death in the untreated group (Figure 4F–H). By contrast, the NA137- and E2-NA137-administered mice were protected from RVFV infection, with a marked decrease in their viral RNAs detected at the time of euthanasia (14 days after the challenge). Compared with PBS group mice, the viral load in the liver of the NA137 group and E2-NA137 group mice was significantly reduced (**** *p* < 0.0001). The above results demonstrate that NA137 and E2-NA137 can efficiently protect mice from the RVFV challenge.

### 3.5. NA137 and E2-NA137 Prevented Infection When Delivered Post-Challenge

**Figure 5:**
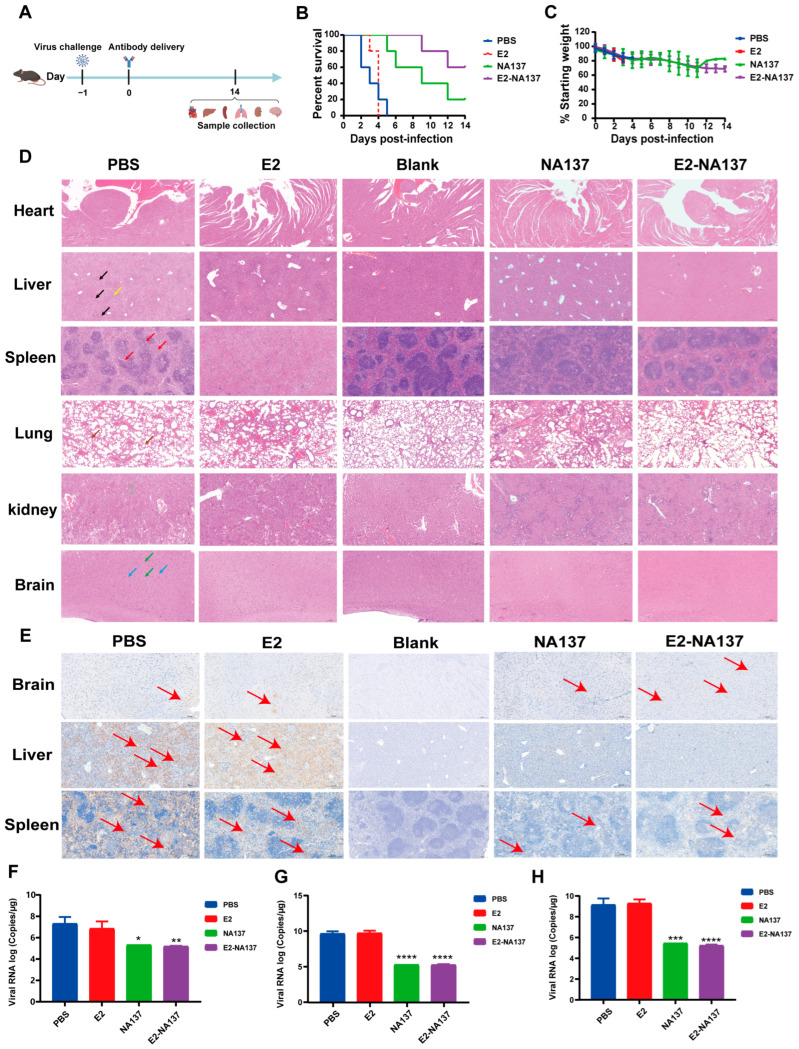
NA137 and E2-NA137 prevented infection when delivered post-challenge. (**A**) Schematic diagram of antibody delivery, virus challenge, sample collection, and immunological assays. (**B**) Mouse mortality and survival curves. (**C**) Body weight changes in mice after RVFV challenge. The tissues from the PBS and E2 groups (at the time of death) and antibody-administrated groups (14 days after the challenge) were collected and analyzed with histopathological and immunohistochemical assays. The Blank group consisted of female A129 mice that had not been challenged with RVFV and that were injected with an equal volume of PBS. (**D**) Hepatocyte necrosis is indicated by black arrows, inflammatory cell infiltration is indicated by yellow arrows, a small amount of cell necrosis in the red pulp is indicated by red arrows, neuronal necrosis is indicated by green arrows, gliocyte proliferation is indicated by blue arrows, alveolar wall capillary congestion is indicated by brown arrows, and necrosis of renal tubular epithelial cells is indicated by gray arrows. Representative histopathology (H&E) of hearts, livers, spleens, lungs, kidneys, and brains in RVFV-infected mice. Scale bar, 200 μm. (**E**) Representative immunohistochemistry (IHC) of brains, livers, and spleens with RVFV Gc-specific monoclonal antibodies. Red arrows indicate RVFV-positive signals. Scale bar, 200 μm. Viral loads in brains (**F**), livers (**G**), and spleens (**H**) of challenged mice were determined via qRT-PCR at the time of death (PBS and E2 groups) or 14 days after the challenge (Blank, NA137, and E2-NA137 groups). Data are displayed as means ± SD. Comparisons based on one-way ANOVA with Dunnett’s multiple comparisons test with * *p* < 0.1, ** *p* < 0.01, *** *p* < 0.001, and **** *p* < 0.0001.

To determine whether NA137 and E2-NA137 had therapeutic activities, the A129 mice were subjected to different antibody treatments using applications 24 h after the virus challenge (Figure 5A). The weight changes (Figure 5C) and survival of the mice were monitored every day (Figure 5B). All animals in the control group died within 5 days of the antibody delivery. As shown in Figure 5B, four mice of the NA137 group died during the antibody application, and the E2-NA137 treatment in RVFV-infected mice *(n* = 5) showed higher efficacy (60% survival) compared with that in the NA137 group (20% survival).

The H&E staining showed significant pathological changes in the mice in the PBS group, including a large number of venous vessels and capillaries on the alveolar wall in the lung tissue, eosinophilic mucus secretion in the local bronchiolar lumen, and a small number of eosinophilic substances in the local alveolar lumen. Large-scale hepatocyte necrosis, nuclear fragmentation or dissolution, structural disorder, punctate lymphocyte infiltration, rare hepatocyte steatosis, and small round vacuoles in the cytoplasm were observed in the liver tissue. In the cerebral cortex and hypothalamus, a small number of neurons could be seen, the cell staining was deepened, the boundary between the nuclei and cytoplasm was unclear, and a small number of capillaries were congested. The number of lymphocytes in the white pulp of the spleen tissue decreased in many places, and scattered lymphocyte necrosis and nuclear fragmentation were seen. A small number of eosinophilic substances were found in the lumina of the renal tubules and sacs in the renal cortex, more tubular epithelial cells were necrotic and exhaled, and nuclear pyknosis was deeply stained. The center muscle fibers of the heart tissue were uniformly colored, the cell boundaries were clear, the shape was consistent, the transverse lines of the cardiomyocytes were clear, light and dark alternated, the interstitium was normal, and no obvious inflammatory cell infiltration was observed. There were also no obvious pathological changes in the E2-NA137 group (Figure 5D). Moreover, the immunohistochemical staining results showed that there were a large number of RVFV-positive signals in the livers, spleens, and brains of the PBS group, while no corresponding positive signals were observed in the E2-NA137 group (Figure 5E).

The viral RNA loads in the brains, livers, and spleens of the PBS group were detected through quantitative RT-PCR, reaching 3.98 × 10^7^ copies/μg RNA, 5.23 × 10^9^ copies/μg RNA, and 2.24 × 10^9^ copies/μg RNA on average at the time of death, respectively. Analyses of the brain and liver tissues, as well as the spleen tissue in necropsied mice, showed significant viral RNA reductions in the combined E2-NA137-treated mice compared with the untreated group (Figure 5F–H). Compared with the PBS group mice, the viral load in the livers and spleens of the E2-NA137 group mice and the livers of the NA137 group mice significantly decreased (**** *p* < 0.0001). These results show that the NA137 and E2-NA137 antibodies provided certain protection for the A129 mice.

### 3.6. AlphaFold 3 Analysis of Binding Epitopes of E2-NA137

**Figure 6:**
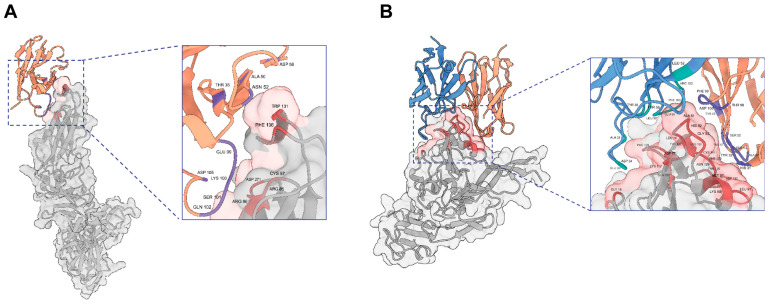
Epitope analysis of E2-NA137. (**A**) Prediction of the interaction between NA137 and Gc using AlphaFold 3. The structure of NA137 is shown in orange, and that of Gc is shown in gray. (**B**) Prediction of the interaction between E2 and Gn using AlphaFold 3. The heavy chain of the E2 antibody is represented in orange, the light chain of the E2 antibody is shown in blue, and the Gn protein is shown in gray.

Our study successfully predicted the three-dimensional structure of the Rift Valley fever virus antigen–antibody complex using AlphaFold 3. The predicted structure was analyzed in depth using ChimeraX 1.8 molecular visualization software. Epitopes were defined as the regions where the distance between the antigen structure surface and antibody structure atoms was ≤3.5 Å, and paratopes were defined as the regions where the distance between the antibody structure surface and antigen structure atoms was ≤3.5 Å. Based on these criteria, we identified the key sites for antibody–antigen interactions. In the complex structures of the Gn antigen and E2 antibody, the epitope amino acid residues involved in antibody binding on the antigen included GLY18, ASP77, LEU79, ALA81, HIS82, GLY83, MET87, LYS88, LEU91, GLN102, LYS117, PRO120, TYR122, THR125, ASP127, ASN129, PHE130, CYS131, and ARG132. The coordinating amino acid residues involved in antigen binding on the antibody heavy chain included HIS31, TYR32, SER52, SER53, ALA54, TYR57, TYR59, THR98, PHE99, and ASP100, and those involved in antigen binding on the antibody light chain included ALA31, GLU33, ASP34, TYR38, LEU52, TYR55, LEU98, GLU99, PHE100, and ARG102. In the complex Gc antigen and NA137 antibody structures, the epitope amino acid residues involved in antibody binding on the antigen included ARG85, ARG86, CYS87, TRP131, PHE136, and ASP271. The coordinating amino acid residues involved in antigen binding on the antibody heavy chain included THR35, ALA50, ASN52, ASP59, GLU99, LYS100, SER101, GLN102, and ASP105 (Figure 6A,B). Further structural analyses of antigen–antibody complexes can be conducted through cryo-electron microscopy in the future.

## 4. Discussion

Rift Valley fever virus is an important pathogen that significantly affects humans and animals, causing high mortality rates and substantial economic losses, especially in the African region. However, in the approximately 90 years since its discovery, no licensed vaccine preparations nor antiviral drugs have been approved for use in humans. Therefore, effective antivirals are urgently needed. However, patients infected with Rift Valley fever virus should be treated with the focus still on supportive treatment at this stage. Drugs mainly include small-molecule chemical drugs such as ribavirin [59] and favipiravir [60,61,62].

A growing number of researchers are now turning their attention to screening for virus-specific neutralizing antibodies, for which the RVFV glycoproteins Gn and Gc, which mediate viral entry and membrane fusion, are considered the crucial antigens. Meng Hao et al. screened and obtained two neutralizing antibodies targeting the Gn protein in rhesus monkeys: 1332F11 and 1331E4 [63]. In 2018, researchers screened three neutralizing antibodies from rabbits immunized with the Gn protein: RV-Gnl, RV-Gn2, and RV-Gn3 [64]. German researchers selected a neutralizing antibody, Gn3, and a binding antibody, Gn32, from mice immunized with the Gn protein in 2020 [65]. Wichgers Schreur PJ et al. first discovered nanobodies targeting the Gn protein in 2020 [57]. As previously reported, six antibodies targeting the Gn protein of Rift Valley fever virus were screened and identified as mAb 1, mAb 2, mAb 3, mAb 4, mAb 5, and mAb 6 [66]. Moreover, Chinese researchers obtained eight neutralizing antibodies aimed at the Gn protein and one neutralizing antibody targeting the Gc protein from a recovered patient of the first imported case of Rift Valley fever in China [28]. Subsequently, a related study showed that five human neutralizing monoclonal antibodies targeting the Gc protein and eleven targeting the Gn protein were selected in 2021 [29]. The above results indicate that most of the currently acquired neutralizing antibodies target the Gn protein of RVFV, while only six neutralizing antibodies target the Gc protein. The Gc protein is more concealed and conserved compared with the Gn protein, making it easier to screen for broad-spectrum neutralizing antibodies. Additionally, we chose to screen neutralizing antibodies targeting Gc antigens in alpaca considering their nanobody characterization, such as high specificity, favorable stability, and the ability to recognize hidden antigen epitopes. In this study, we isolated one NAb (NA137) against Gc with an IC_50_ value of 2.701 μg/mL from an alpaca. Comparing the Gc amino acid sequences of MP-12 with other RVFVs showed a high degree of homology (Figure S5). The high conservation of the RVF genome sequence suggests either that the overall tolerance for mutation within the RVF virus genome is very low or that the viruses in this group have a relatively recent common ancestor [67]. RVFVs have low overall molecular evolutionary rates, low overall nucleotide diversity, and low genetic diversity [67]. These results suggest that NA137 may have a broad-spectrum neutralization effect on other RVF viruses. It has previously been reported that 326 is a neutralizing antibody targeting the Gc protein of the Rift Valley fever virus. Using the Vero E6 cell model, the IC_50_ value of 326 was 11.11 μg/mL, and the IC_50_ value of NA137 was 2.701 μg/mL. The neutralizing activity of the NA137 antibody was superior to that of the 326 antibody. There is currently no study reporting on the in vivo protective effect of the 326 antibody. In the preventive administration mode, the survival rate of mice with NA137 antibodies was 80%, and in the therapeutic administration mode, the survival rate of the mice was 20%. The above results indicate that NA137 is a promising candidate drug for treating patients with Rift Valley fever.

To further enhance the neutralizing activity of antibodies, three bispecific antibodies in IgG-VHH format were constructed, with the high-affinity E2 antibody targeting the Gn protein as the parental antibody, and the results showed that the neutralizing ability was improved significantly, with the IC_50_ values of E2-NA137, E2-NA19, E2-NA220 being 0.010, 0.023, and 0.012, respectively. Even though E2 has no neutralizing activity in vitro, it significantly increases the neutralizing potency of NA137. This study is the first trial on bispecific antibodies in the IgG-VHH format. The binding antibody targeting the Gn protein was selected as the parental antibody, and the VHH targeting the Gc protein was fused with a linker.

To determine whether NA137 and E2-NA137 have therapeutic activities, A129 mice inoculated with the RVFV rMP12 strain were subjected to different antibody treatments, with applications 24 h before or after virus challenge studies being carried out. In the pre-exposure setting, the antibody treatment exhibited an 80% survival rate in the NA137 and E2-NA137 groups. The treatment of RVFV-infected mice with antibodies showed a certain efficacy (20% and 60% survival rates in the NA137 and E2-NA137 groups, respectively) in the post-exposure setting. Additionally, E2-NA137 can simultaneously target Gn and Gc proteins and prevent immune escape. Compared with NA137 (IC_50_ = 2.701 μg/mL), the neutralizing activity of E2-NA137 (IC_50_ = 0.010 μg/mL) was enhanced. The results indicate that compared with NA137, E2-NA137 is more protective against the RVFV challenge. Moreover, E2-NA137 provides better protection when administered prior to the challenge. At present, there are no reports on the structure of the Gc protein–antibody complex. In this study, we attempted to analyze the structure of the Gc-NA137 antigen–antibody complex using cryo-electron microscopy. Unfortunately, it was difficult to continue advancements in the 2D stage; we are still improving the preparation method of the compound for further study. We further analyzed the neutralizing antibodies against the Rift Valley fever virus reported in the current literature. As shown in Table 1, most neutralizing antibodies targeted the RVFV Gn protein, with only six neutralizing antibodies targeting the RVFV Gc protein. Among these six neutralizing antibodies, the only reported neutralizing antibody targeting the Gc protein, R5, had a protection rate of only 25% in Balb/c mice [28]. The above results suggest that the E2-NA137 antibody may be a candidate drug for the treatment of patients with Rift Valley fever.

Gn is a type I transmembrane protein that forms together with glycoprotein Gc non-covalently linked heterodimers on the lipid bilayer envelope of the virion and allows for virus attachment, uptake into cells, and Gc protein-mediated cell fusion. It constitutes a primary target of the nAb response generated during both natural infection and immunization [22,23,68,69]. We therefore hypothesize that the mechanism is based on the binding of E2 to the glycoprotein Gn, which induces a conformational change and makes the epitope of NA137 more accessible. Moreover, both SFTSV and RVFV belong to the Phenuiviridae family in different genera, and previous studies have demonstrated that their glycoproteins display similar overall structures [23]. Based on this structural analysis, we propose that E2-NA137 binds to the Gn head in the prefusion stage, and then Gc undergoes pH-dependent rearrangements, which drives Gn–Gc dissociation, thereby exposing the epitope of NA137 on Gc domain II. E2-NA137 can bind to both Gn and Gc in the intermediate stage, which blocks the fusion process, similar to BsAb3 reported in the previous literature [70]. Therefore, further detailed studies are needed to elucidate the molecular E2-NA137 neutralization mechanism through cryo-electron microscopy.

## 5. Conclusions

In summary, we obtained 91 binding antibodies targeting the Gc protein through phage display technology. Among them, one nanobody (NA137) with high neutralizing activity and two nanobodies (NA19 and NA220) with weak neutralizing activity were screened. Subsequently, three bispecific antibodies, E2-NA137, E2-NA19, and E2-NA220, were constructed, among which E2-NA137 had a good protective effect in vitro. This protective effect was further evaluated in A129 mice, and the results show that E2-NA137 provides certain protection against the RVFV challenge in vivo. This study provides a new therapeutic idea for treating Rift Valley fever virus infection and has important reference value for the development of drugs for the prevention and treatment of this virus.

## Figures and Tables

**Table 1:** Research status of neutralizing antibodies against Rift Valley fever virus. * *p* < 0.1, ** *p* < 0.01.

Name	Target	Source	In Vitro Efficacy (IC_50_)	In Vivo Efficacy	Ref.
RV-Gn1	Gn	Rabbit	3000 ng/mL (Vero)	BALB/c mice (100%)	[64]
RV-Gn2	Gn	Rabbit	2800 ng/mL (Vero)	BALB/c mice (100%)	[64]
RV-Gn3	Gn	Rabbit	2100 ng/mL (Vero)	BALB/c mice (100%)	[64]
Gn3	Gn	Mouse	33,000 ng/mL (Vero 76)	BALB/c mice (58.3%)	[65]
Gn3 + Gn32	Gn	Mouse	24,600 ng/mL (Vero 76)	BALB/c mice (100%)	[65]
268	Gn	Human	0.1 ng/mL (Vero)	C57BL/6 mice (90%)	[29]
142	Gn	Human	5.9 ng/mL (Vero)		[29]
436	Gn	Human	2.5 ng/mL (Vero)		[29]
429	Gn	Human	0.9 ng/mL (Vero)		[29]
379	Gn	Human	1.3 ng/mL (Vero)		[29]
140	*	Human	1.1 ng/mL (Vero)	C57BL/6 mice (100%)	[29]
426	Gn	Human	9.1 ng/mL (Vero)		[29]
296	Gn	Human	638 ng/mL (Vero)		[29]
220	*	Human	157 ng/mL (Vero)		[29]
250	Gc	Human	51 ng/mL (Vero)		[29]
401	Gn	Human	2120 ng/mL (Vero)		[29]
128	Gc	Human	38 ng/mL (Vero)		[29]
121	Gc	Human	35 ng/mL (Vero)		[29]
326	Gc	Human	70 ng/mL (Vero)		[29]
144	*	Human	1.9 ng/mL (Vero)		[29]
381	Gn	Human	4435 ng/mL (Vero)		[29]
226	Gn	Human	7692 ng/mL (Vero)	C57BL/6 mice (50%)	[29]
229	NT **	Human	3040 ng/mL (Vero)		[29]
405	Gn	Human	>(Vero)		[29]
249	Gc	Human	6687 (Vero)		[29]
mAb 1	Gn	Mouse	28 ng/mL (Vero-E6)	C57BL/6 mice (100%)	[66]
mAb 2	Gn	Mouse	1532 ng/mL (Vero-E6)	C57BL/6 mice (100%)	[66]
mAb 3	Gn	Mouse	>(Vero-E6)	C57BL/6 mice (100%)	[66]
mAb 4	Gn	Mouse	>(Vero-E6)		[66]
mAb 5	Gn	Mouse	>(Vero-E6)		[66]
mAb 6	Gn	Mouse	>(Vero-E6)		[66]
RVFV-VHHs	Gn	Alpaca	(Vero-E6)	BALB/c mice (20%)	[57]
1332F11	Gn	Rhesus monkey	170 ng/mL (Huh7)		[63]
1331E4	Gn	Rhesus monkey	340 ng/mL (Huh7)		[63]
R4	Gn	Human	46.8 ng/mL (Vero)	BALB/c mice (100%)	[28]
R5	Gc	Human	1370 ng/mL (Vero)	BALB/c mice (20%)	[28]
R12	Gn	Human	1.85 ng/mL (Vero)	BALB/c mice (100%)	[28]
R13	Gn	Human	56.2 ng/mL (Vero)	BALB/c mice (100%)	[28]
R15	Gn	Human	0.53 ng/mL (Vero)	BALB/c mice (100%)	[28]
R16	Gn	Human	0.29 ng/mL (Vero)	BALB/c mice (100%)	[28]
R17	Gn	Human	2.53 ng/mL (Vero)	BALB/c mice (100%)	[28]
R19	Gn	Human	51.1 ng/mL (Vero)	BALB/c mice (100%)	[28]
R22	Gn	Human	73.7 ng/mL (Vero)	BALB/c mice (100%)	[28]

## Data Availability

Data are contained within the article and Supplementary Materials.

## Supplementary Materials

The following supporting information can be downloaded at https://www.mdpi.com/article/10.3390/v17040559/s1, Figure S1: Analysis of binding activity of nanobodies to Gc protein. Figure S2: Binding activity of antibodies to Gc (EC_50_). Figure S3: Length distribution of CDR3. The left figure is a length histogram. Figure S4: Protection efficacy of monoclonal antibodies against RVFV infection in Vero E6 cells (partial result presentation). Figure S5: Homology analysis of amino acid sequence of Gc.
