# Supplementary figures and images for: Screening of Neutralizing Antibodies Targeting Gc Protein of RVFV
Source: Viruses. 2025 Apr 12;17(4):559. doi: 10.3390/v17040559 (PMC12031069; doi:10.3390/v17040559)

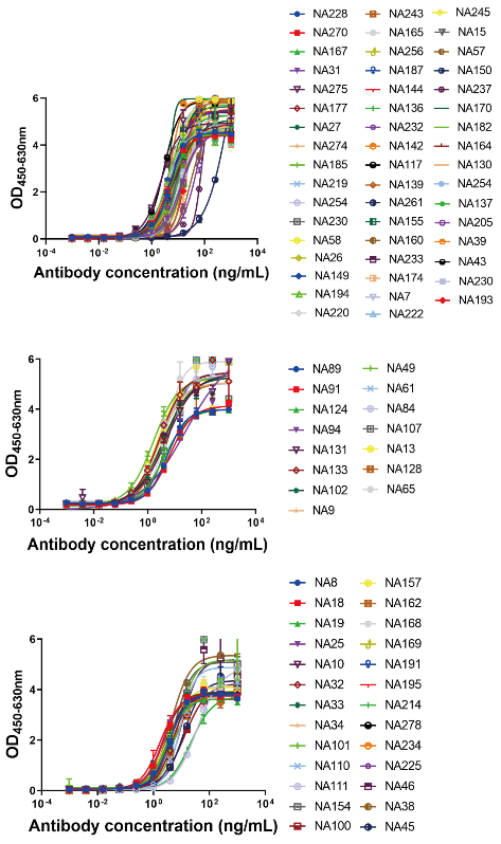

Supplement: Supplementary file 1 [file viruses-17-00559-s001.zip › Figure S1.tif]

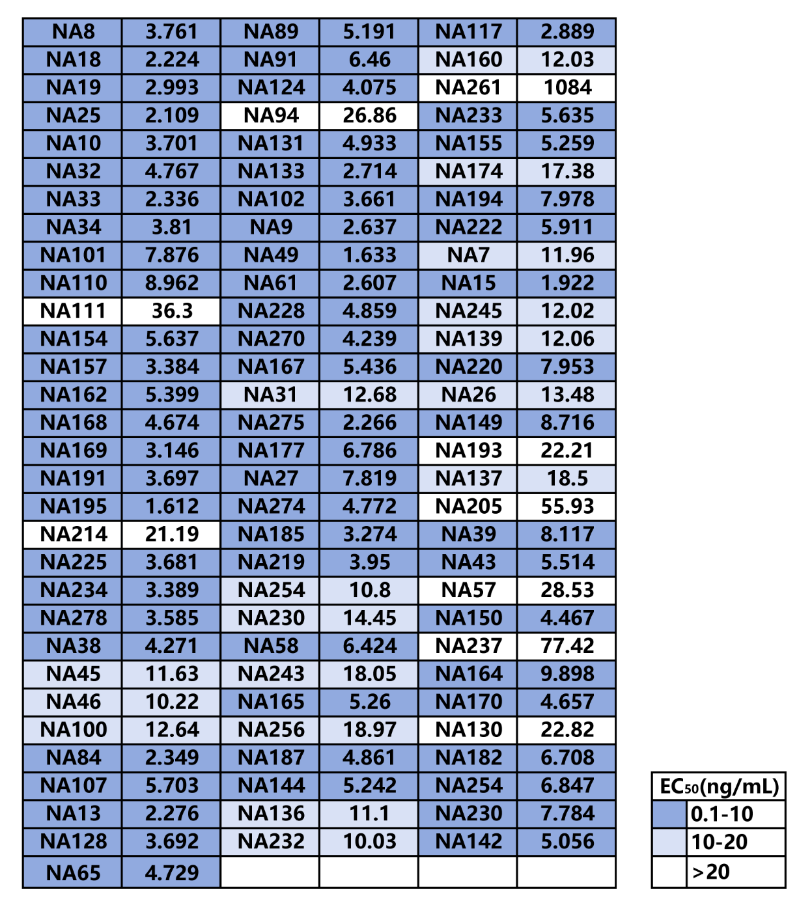

Supplement: Supplementary file 1 [file viruses-17-00559-s001.zip › Figure S2.tif]

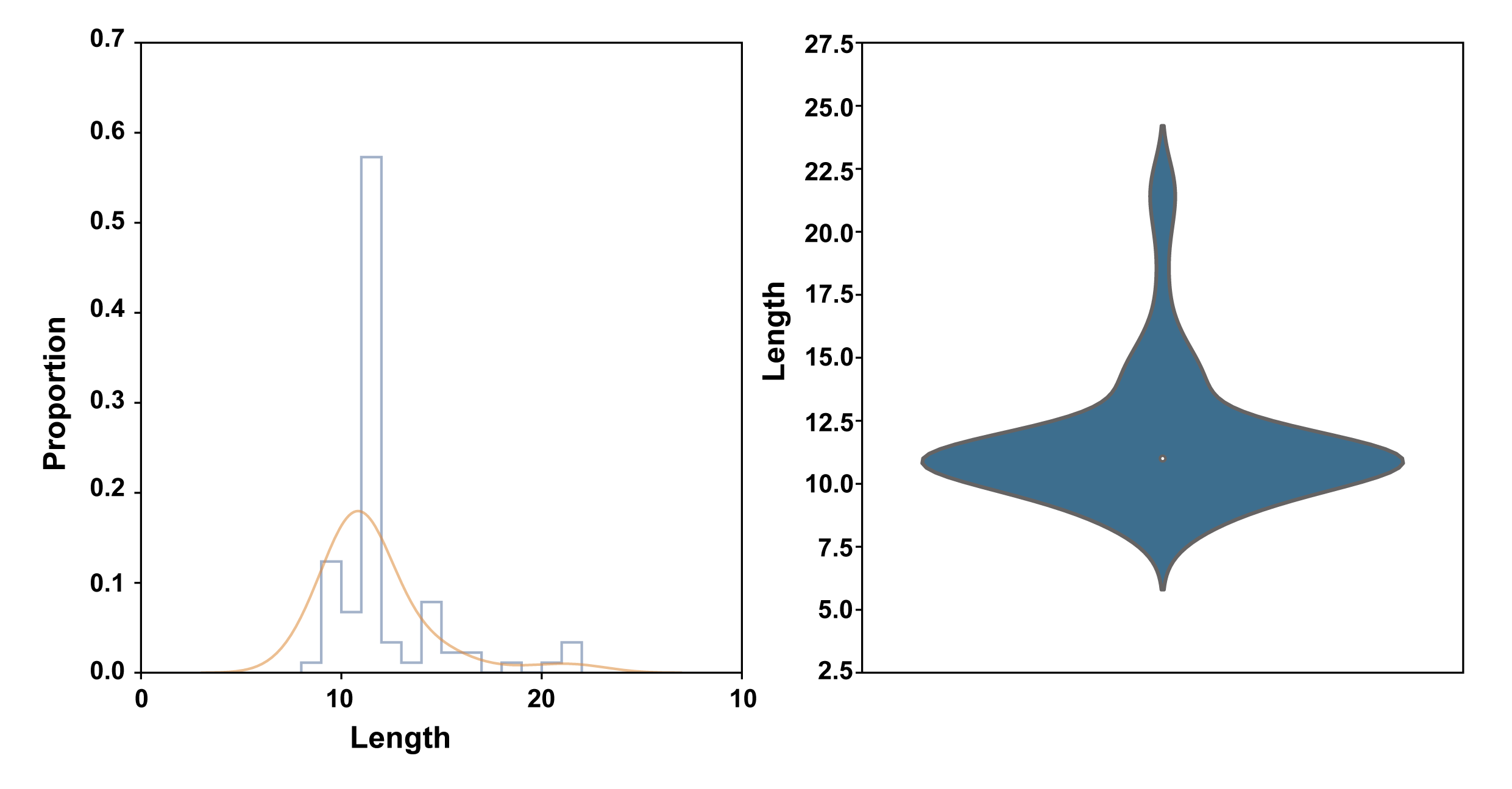

Supplement: Supplementary file 1 [file viruses-17-00559-s001.zip › Figure S3.tif]

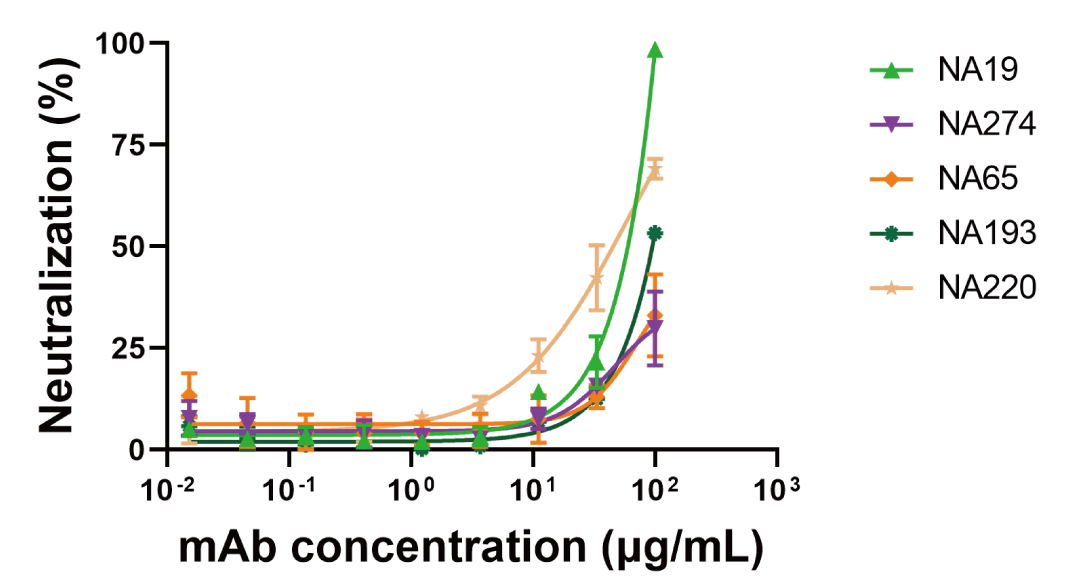

Supplement: Supplementary file 1 [file viruses-17-00559-s001.zip › Figure S4.tif]

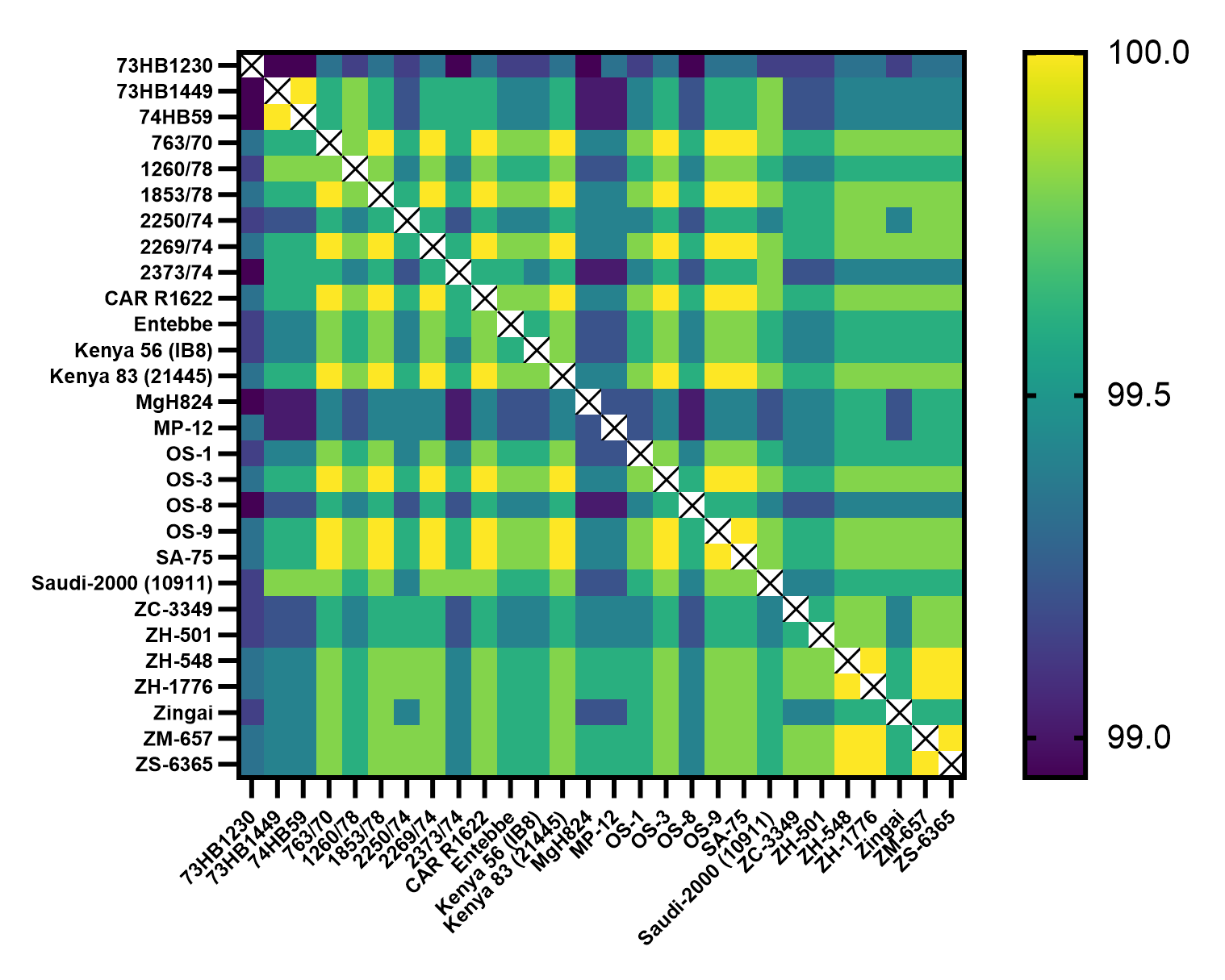

Supplement: Supplementary file 1 [file viruses-17-00559-s001.zip › Figure S5.tif]
